# An integrative approach to the study of *Helicotylenchus* (Nematoda: Hoplolaimidae) Colombian and Brazilian populations associated with *Musa* crops

**DOI:** 10.21307/jofnem-2020-054

**Published:** 2020-07-06

**Authors:** Donald Riascos-Ortiz, Ana Teresa Mosquera-Espinosa, Francia Varón De Agudelo, Claudio Marcelo Gonçalves de Oliveira, Jaime Eduardo Muñoz-Florez

**Affiliations:** 1 Facultad de Ciencias Agropecuarias, Universidad Nacional de Colombia, Palmira-Colombia, Programa de Agronomía, Universidad del Pacífico, Buenaventura, Colombia; 2 Departamento de Ciencias Naturales y Matemáticas, Pontificia Universidad Javeriana, Cali, Colombia; 3 Centro de Investigación en Palma de Aceite-Cenipalma, Bogotá, Colombia; 4 Laboratorio de Nematologia, Instituto Biológico, Campinas, São Paulo, Brasil; 5 Facultad de Ciencias Agropecuarias, Universidad Nacional de Colombia, Palmira, Colombia

**Keywords:** Banana, *Helicotylenchus*, *H. multicinctus*, *H. dihystera*, *H. erythrinae*, *H. californicus*, Plantain, Taxonomy

## Abstract

In total, 10 populations of *Helicotylenchus* associated with *Musa* spp., six from Colombia, and four from Brazil were identified to the species level. Morphological and morphometric data were recorded for each population, performed a principal components analysis (PCA), a conglomerate, along with consensus sequences of D2 to D3 expansion segment of the large subunit of ribosomal DNA (28S) for different populations. Identify of species were performed using the basic local alignment search tool (BLAST), and the evolutionary relationships of species were explored using a phylogeny study. Four species of *Helicotylenchus* were identified based on morphological and morphometric data: *H. multicinctus, H. dihystera, H. erythrinae*, and *H. californicus*. PCA and conglomerate analysis clearly separated these species. BLAST and phylogenetic analysis confirmed the presence of these species associated with *Musa* spp. crops. This is the first report of these species in Colombia through integrative taxonomy.

*Helicotylenchus* species are ecto and semiendoparasitic nematode, with worldwide distribution and with great importance in crops of *Musa* spp., as it causes the highest losses of production and yield after *Radopholus similis*, *Pratylenchus* spp., and *Meloidogyne* spp. (Araya and De Waele, 2004; Karakas, 2007; Singh et al., 2013; Ravichandra, 2014). The most limiting *Helicotylenchus* species in banana and plantain crops around the world are *H. multicinctus* (Golden, 1956), *H. dihystera* (Sher, 1961), and *H. erythrinae* (Golden, 1956). However, other species of *Helicotylenchus* have been found to be associated with *Musa* spp. crops in minor frequency. These species include *H. abunaamai* (Siddiqi, 1972), *H. incisus*, *H. africanus*, *H. punicae*, *H. egyptiensis*, *H. variocaudatus* (Fortuner, 1984), and *H. digonicus* (Perry et al., 1959; Castillo and Gómez-Barcina, 1993; Van Den Berg et al., 2003; Khan and Hasan, 2010; Ravichandra, 2014).

These species occur individually or in a mixture associate with Musaceae of different production zones in the world, including Vietnam, Venezuela, Democratic Republic of Congo, Brazil, South Africa, India, Mexico, and Greece. These nematodes cause injuries to outer layers of cortical tissue (observed as brown–reddish to black discrete spots), as well as disruption and progressive deterioration of the roots system and, as a consequence, the plant’s capacity for the uptake of water and nutrients is affected negatively (Orbin, 1973; Ngoc Chau et al., 1997; Crozzoli, 2009; Dias-Arieira, 2010; Khan and Hasan, 2010; Kamira et al., 2013; Roy et al., 2014; Daneel et al., 2015; Lara et al., 2016; Tzortzakakis et al., 2017). In addition, bunch maturation and size are reduced, with production losses between 19 and 34% at 2 to 3 years after planting, although the damage may be greater when *R. similis* is absent (McSorley and Parrado, 1986; Barekye et al., 2000; Guzmán-Piedrahita, 2011; Selvaraj et al., 2014).

Production losses in plantain and banana by *Helicotylenchus* spp. depend on two main factors. On the one side, the susceptibility of planting material and nematode density, because differences in the population level of *H. multicinctus* have been registered among *Musa* spp. cultivars with a high correlation between its density and the necrotic and dead roots percentage (Speijer and Ssango, 1999). On the other hand, losses depend on environmental conditions, since *Helicotylenchus* spp. are predominant in crops cultivated between 1,000 and 1,350 meters above sea level in andisols and vertisols soils with high contents of Ca, P, Mn and reduced organic matter (Speijer and Ssango, 1999; Karakas, 2007; Araya et al., 2011).

Although *Helicotylenchus* has been reported in the banana and plantain crops of Colombia and Brazil, morphological, morphometric, and molecular data are scarce for these geographical regions (Zuñiga et al., 1979; Villegas, 1989; Guzmán-Piedrahita and Cataño-Zapata, 2004; Torrado-Jaime and Castaño-Zapata, 2009). In order to contribute to the knowledge of the taxonomic identity of the species of *Helicotylenchus* associated with Musaceae in Colombia and Brazil, the present study has the following objectives: (i) to identify the species of *Helicotylenchus* that are associated with banana and plantain crops, using a combination of morphological, morphometric, and molecular analysis, and (ii) to elucidate the phylogenetic relationships of *Helicotylenchus* species that are associated with banana and plantain crops in the departments of Quindío, Risaralda y Valle del Cauca in Colombia and Minas Gerais in Brazil.

## Materials and methods

### Area of study, sampling, and nematodes extraction

Root and soil samples were collected from banana and plantain rhizosphere crops in the departments of Quindío (Calarcá and Córdoba municipalities), Risaralda (La Celia municipality), and Valle del Cauca (Palmira and Buenaventura municipalities) in Colombia and in Janauba, Minas Gerais state, Brazil during 2015 to 2018. In each crop, compound samples were collected, comprising 15 to 20 plants/ha. Nematodes were extracted from roots and soil using a modification of Cobb’s method (Ravichandra, 2014). Afterwards, representative populations of *Helicotylenchus* were selected for posterior analysis in the microbiology and molecular biology laboratories of the National University of Colombia (Palmira, Valle del Cauca, Colombia) and the Laboratory of Nematology of the Biological Institute (Campinas, São Paulo, Brazil).

### Morphologic and morphometric analysis

Nematodes extracted and identified as *Helicotylenchus* were killed with heat at 60°C for 4 min and fixated in 2% formalin. Semipermanent preparations were made and morphometric data were registered following Boag and Shamim Jairajpuri (1985) and Uzma et al. (2015). Microphotographs were taken using a compound microscope equipped with differential interference contrast–DIC (DM2500, Leica, Germany).

### Statistical analysis:

Morphometric data were analyzed using the Community Analysis Package (PISCES Conservation Ltd, Lymington UK, 1995) with principal components analysis (PCA) and conglomerate analysis to determine groupings and evaluate those characters that could discriminate species.

### Extraction, amplification, and DNA sequencing

For molecular analysis, DNA was extracted by the Proteinase K method of Múnera et al. (2009) with modifications. A single nematode was crushed with a sterile scalpel and transferred to an Eppendorf tube with 15 μl of worm lysis buffer (50 mM KCl, 10 mM Tris pH 8.0, 15 mM MgCl2, 0.5% Triton x–100, 4.5% Tween–20, 0.09% Proteinase K). The tubes were incubated at −80°C (15 min), 65°C (1 h), and 95°C (15 min), centrifuged to 16,000 g (1 min) and stored at −20°C. Amplification of D2 to D3 expansion segment of the large subunit – LSU of ribosomal DNA (28S) was done using forward primer D2A (5′–ACAAGTACCGTGAGGGAAAGTTG–3′) and reverse D3B (5′–TCCTCGGAAGGAACCAGCTACTA–3′) (De Ley et al., 1999). The PCR conditions were initial denaturation during 2 min at 94°C followed by 40 cycles of 45 s at 94°C, 45 s at 55°C, and 1 min at 72°C and final extension of 10 min at 72°C. PCR products were sequenced in both directions at BIONNER Korea.

### Phylogenetic analysis

Consensus sequences were edited using the software Geneious (Kearse et al., 2012) and BLAST (Basic Local Alignment Search Tool) at NCBI (National Center for Biotechnology Information) was used to confirm the species’ identity of the sequences. To estimate the phylogenetic history of *Helicotylenchus,* D2 to D3 sequences of others specimens of the species were downloaded from GenBank, including a sequence of *Rotylenchus magnus*, which was used as outgroup. Sequence alignment was performed using MAFFT v7 (Katoh et al., 2002) (protocol Q-INS-i), and jModelTest v2.1.7 (Posada, 2008) to find the best nucleotide substitution model, based on the Akaike information criterion corrected for small sample sizes. Afterwards maximum likelihood (ML) was used to estimate a tree with 250 bootstraps and the general time reversible model with allowance for gamma distribution of rate variation (GTR+Γ) in RAxML v8 (Stamatakis, 2014). The phylogeny of *Helicotylenchus* was inferred using MrBayes v3.2.6 (Ronquist et al., 2012) with the GTR + Γ model. Two independent Metropolis-coupled Markov chain Monte Carlo (MCMCMC) searches were performed for 2 million generations sampling every 2,000 steps. Convergence was assessed using Tracer v1.5 (burn-in = 20% of the samples), and by examining the average standard deviation of split frequencies among parallel chains. The consensus tree was calculated from the posterior distribution of 1,600 phylogenies. The Bayesian analysis was performed in the CIPRES Science Gateway (Miller et al., 2010).

## Results

### Morphological and morphometric identification of nematodes

Four species of *Helicotylenchus* were identified in the study areas: *H. multicinctus*, *H. dihystera*, *H. erythrinae*, and *H. californicus*. Morphological and morphometric data from each species closely resembled type and reference populations species (Tables 1-4).

**Table 1.** Morphometric data of *H. multicinctus* and reference populations.

Species	*H. multicinctus*	*H. multicinctus*	*H. multicinctus*	*H. multicinctus*	*H. multicinctus*	*H. multicinctus*
Locality/publication	Plantain, Cordoba–Quindío–HM1 *n* = 19	Plantain, Rozo–Palmira–HM2 *n* = 20	Banana, Brazil HM3–* *n* = 24	Banana, Brazil 233–HM4 *n* = 21	Goodey (1940), Golden (1956) *n* = 2	Uzma et al. (2015) *n* = not defined
L	0.6 ± 0.1 (0.4–0.8)	0.6 ± 0.04 (0.5-0.7)	0.5 ± 0.05 (0.5-0.6)	0.5 ± 0.1 (0.4-0.6)	0.54 (0.46-0.68)	0.47-0.53
a	26.1 ± 2.8 (22.5-31.1)	24.4 ± 1.5 (22.1-27.4)	24.7 ± 1.9 (22.4-30.3)	24.5 ± 2.0 (21.2-27.9)	24.47 (23.8-28.5)	24.0-30.0
c	45.6 ± 5.1 (36.1-55.2)	44.6 ± 5.6 (36.5-59.8)	49.2 ± 7.0 (37.7-64.1)	50.7 ± 7.4 (39.6-69.6)	53.8 (48.0-63.0)	35.0-46.0
c′	0.9 ± 0.1 (0.7-1.1)	0.9 ± 0.1 (0.7-1.0)	0.8 ± 0.1 (0.6-1.1)	0.8 ± 0.1 (0.4-1.0)	0.8-1.0	0.8-1.0
V%	68.4 ± 1.6 (65.9-71.7)	67.2 ± 1.7 (64.1-70.0)	68.9 ± 2.3 (65.0-73.1)	69.1 ± 1.1 (66.2-71.1)	69.0 (64.6-71.8)	65.0-69.0
DGO	8.6 ± 0.6 (8.0-10.0)	9.5 ± 0.5 (9.0-10.0)	9.1 ± 1.0 (7.0-11.0)	9.3 ± 1.1 (7.0-11.0)	–	–
Stylet length	23.4 ± 1.3 (21.0-26.0)	23.7 ± 0.7 (23.0-25.0)	23.5 ± 0.9 (22.0-25.0)	23.1 ± 0.8 (22.0-24.0)	20.0-24.0	22.0-24.0
Number of head annuli	4.0 ± 0.6 (3.0-5.0)	4.0 ± 0.5 (4.0-5.0)	4.0 ± 0.5 (4.0-5.0)	4.0 ± 0.0 (4.0-4.0)	3.0-5.0	3.0-4.0
Tail length	13.0 ± 1.9 (10.0-16.0)	12.9 ± 1.2 (10.0-15.0)	11.3 ± 1.7 (8.0-15.0)	10.5 ± 1.9 (7.0-15.0)	–	–
Number of tail annuli	9.0 ± 1.2 (7.0-12.0)	9.3 ± 1.3 (7.0-12.0)	8.0 ± 1.9 (5.0-12.0)	7.7 ± 1.5 (5.0-10.0)	6.0-13.0	6.0-12.0
Position of phasmid	3.0 ± 1.2 (2.0-6.0)	3.8 ± 1.0 (3.0-6.0)	5.0 ± 2.1 (2.0-7.0)	5.0 ± 1.2 (3.0-7.0)	1.0-6.0	2.0-6.0

**Note:** Measurements in μm; mean ± s.d. (range), except for L in mm.

**Figure 1:**
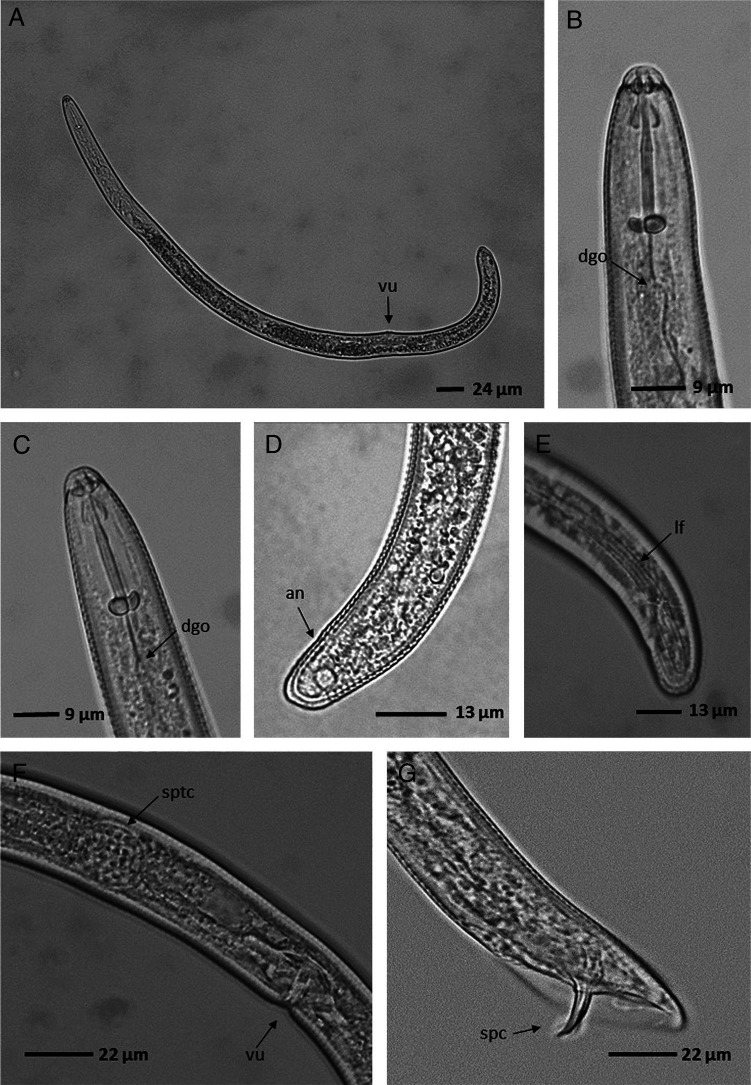
*Helicotylenchus multicinctus*. A: Habitus post-mortem of a female, B and C: Anterior region of female body, D and E: Posterior region of female body, F: Medium region of female body, G: Posterior region of male body. vu = Vulva, dgo = Dorsal esophageal Gland Orifice, an = Anus, lf = Lateral field, sptc = Spermatheca, spc = Spicule.

*Helicotylenchus multicinctus* populations were identified in plantain crops of Colombia (Córdoba, Quindío and Rozo, Valle del Cauca) and banana crops of Brazil (Minas Gerais). Morphologically, these populations show a habitus post-mortem open C form, a hemispherical cephalic region, the shape of the stylet knobs flattened anteriorly and rounded posteriorly, a rounded tail, functional spermatheca and males present (Fig. 1, Table 1).

**Figure 2:**
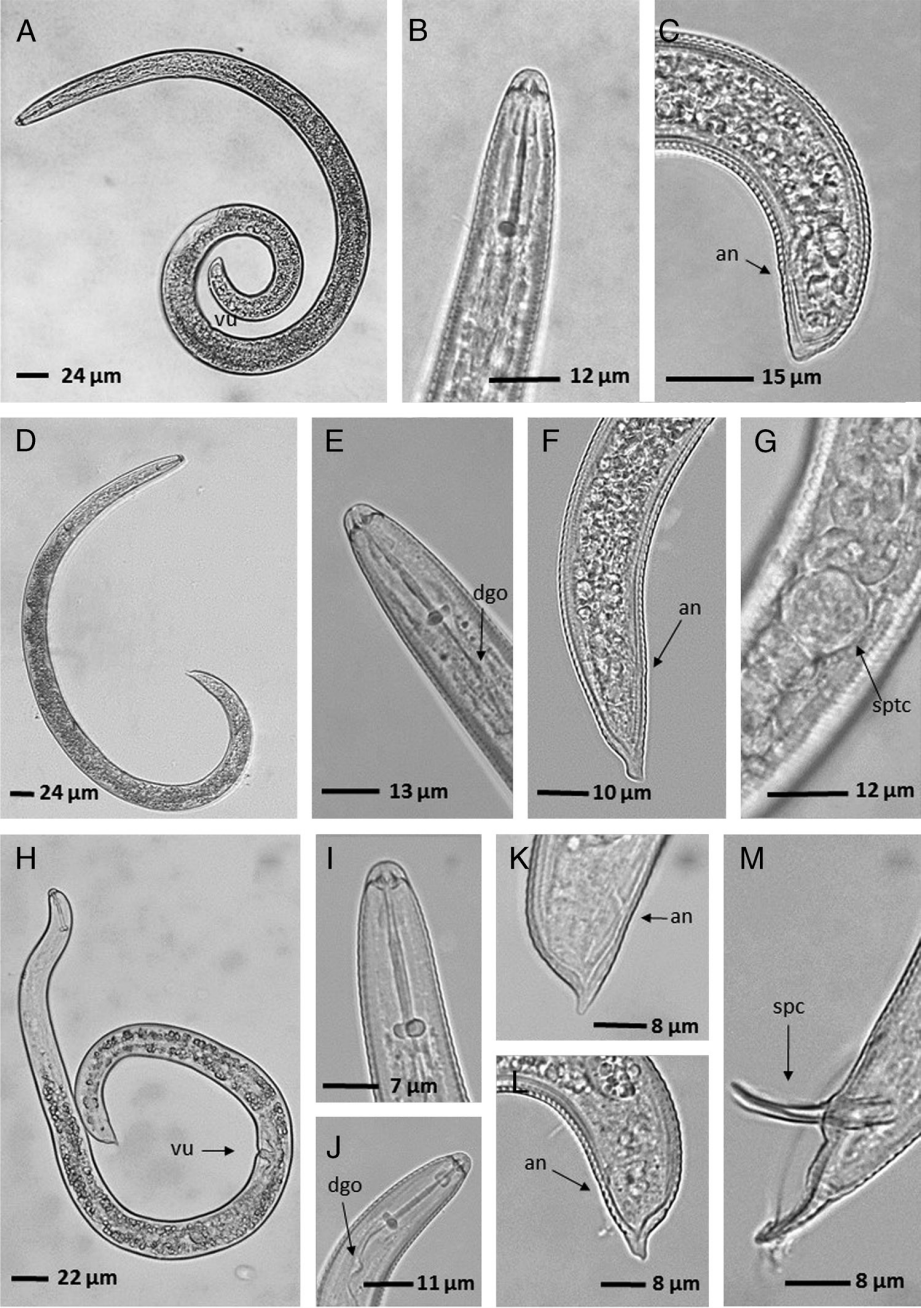
*Helicotylenchus dihystera, H. erythrinae*, and *H. californicus*. A: Habitus post mortis of a female in *H. dihystera*, B: Anterior region of female body in *H. dihystera*, C: Posterior region of female body in *H. dihystera*, D: Habitus post mortis of a female in *H. erythrinae*, E: Anterior region of female body in *H. erythrinae*, F: Posterior region of female body in *H. erythrinae*, G: Spermatheca of female in *H. erythrinae,* H: Habitus post mortis of female in *H. californicus*, I and J: Anterior region of female body in *H. californicus*, K: Posterior region of female body in *H. californicus* with short ventral projection and sharply pointed tail, L: Posterior region of female body in *H. californicus* with short ventral projection and blunt tail, M: Posterior region of male body in *H. californicus.*

Populations of *H. dihystera* were founded in plantain and banana crops from Colombia (Córdoba, Quindío). Morphologically, the populations of this species show a habitus post-mortem with a spiral shape, a hemispherical cephalic region with the shape of stylet knobs indented or flattened anteriorly and rounded posteriorly, a conoide tail and males absent (Fig. 2, Table 2).

**Table 2.** Morphometric data of *H. dihystera* and reference populations.

Species	*H. dihystera*	*H. dihystera*	*H. dihystera*	*H. dihystera*	H. dihystera	
Locality/publication	Banana, Córdoba–Quindío–HD1	Plantain, Córdoba–Quindío–HD2	Sher (1961)	Uzma et al. (2015)	Wount and Yates	
	*n* = 10	*n* = 15	*n* = 12	*n* = not defined	*n* = 10	*n* = 10
L	0.62 ± 0.08 (0.53-0.77)	0.61 ± 0.06 (0.48-0.69)	0.67 (0.61-0.86)	0.59-0.79	0.67 ± 0.05 (0.57-0.73)	0.71 ± 0.04 (0.61-0.75)
a	25.5 ± 1.94 (22.93-29.69)	24.0 ± 1.83 (20.50-28.13)	29.5 (26.0-34.0)	27-35	29.3 ± 2.21 (26.0-33.0)	29.5 ± 1.35 (27.0-32.0)
c	43.8 ± 8.36 (25.12-55.45)	45.3 ± 4.49 (39.0-56.3)	48.0 (40.0-65.0)	35- 49	46.0 ± 4.69 (37.0-53.0)	43.0 ± 4.67 (35.0-50.0)
c′	1.0 ± 0.17 (0.80-1.31)	0.9 ± 0.07 (0.71-0.94)	1.0-1.3	0.8-1.2	1.11 ± 0.11 (1.0-1.3)	1.09 ± 0.11 (0.9-1.3)
V%	64.6 ± 1.19 (62.24-66.35)	64.5 ± 1.24 (61.94-66.12)	63.0 (60.0-66.0)	60-65	65 ± 1.32 (63.0-67.0)	63.0 ± 0.84 (61.0-64.0)
DGO	14.7 ± 1.38 (13.0-16.0)	14.9 ± 1.57 (13.0-17.0)	–	–	11.5 ± 1.63 (8.5-13.5)	10.0 ± 1.25 (7.0-11.0)
Stylet length	24.4 ± 0.97 (23.0-26.0)	23.8 ± 0.77 (23.0-25.0)	26.0 (24.5-27.5)	24-29	27.9 ± 1.08 (25.5-29.0)	26.1 ± 0.74 (25.0-27.0)
Number of head annuli	4.0 ± 0.0 (4.0-4.0)	4.0 ± 0.26 (4.0-5.0)	3.0-4.0	4-5	–	–
Tail length	14.5 ± 3.06 (11.0-21.0)	13.5 ± 1.51 (12.0-16.0)	–	–	14.5 ± 1.48 (12.0-17.0)	16.7 ± 2.50 (12.0-21.0)
Number of tail annuli	8.0 ± 1.88 (5.0-12.0)	8.0 ± 0.68 (7.0-10.0)	8-12	6-12	–	–
Position of phasmid	8.0 ± 0.99 (7.0-10.0)	9.0 ± 1.91 (6.0-11.0)	6-12	5-11	–	–

**Note:** Measurements in μm; mean ± s.d. (range), except for L in mm.

The populations identified as *H. erythrinae* occurred in Colombian plantain and banana crops (Calarcá, Quindío and La Celia, Risaralda). Morphologically, the populations of this species show a habitus post-mortem of a loose spiral, a hemispherical cephalic region with the shape of stylet knobs indented or flattened anteriorly and rounded posteriorly, a tail with a long ventral projection, females with functional spermatheca and males present (Fig. 2, Table 3).

**Table 3.** Morphometric data of *H. erythrinae* and reference populations.

Species	*H. erythrinae*	*H. erythrinae*	*H. erythrinae*	*H. erythrinae*	*H. erythrinae*	
Locality/publication	Banana, Calarcá–Quindío (La Julia)–HE1	Plantain, Calarcá –Quindío (Resta)–HE2	Van Den Berg and Heyns (1975)	Uzma et al. (2015)	Wount and Yates	
	*n* = 15	*n* = 25	*n* = 7	*n* = not defined	*n* = 10	*n* = 10
L	0.53 ± 0.1 (0.4-0.7)	0.6 ± 0.1 (0.46-0.68)	0.5 (04-0.6)	0.48-0.61	0.87 ± 0.06	0.69 ± 0.05
a	25.03 ± 2.0 (22.7-29.7)	25.90 ± 2.2 (22.86-30.91)	25.1 (21.1-26.7)	23.0-26.0	29.7 ± 1.64 (27.0-32.0)	28.2 ± 1.87 (24.0-30.0)
c	25.93 ± 4.1 (19.2-31.5)	28.09 ± 3.0 (21.82-32.94)	28.9 (23.3-30.3)	27.0-34.0	43.0 ± 5.99 (31.0 -51.0)	48.0 ± 10.55 (35.0-74.0)
c′	1.46 ± 0.2 (1.1-1.7)	1.41 ± 0.15 (1.13-1.73)	1.4 (1.3-1.4)	1.0-1.6	1.13 ± 0.106 (1.0-1.3)	1.03 ± 0.206 (0.60-1.30)
V%	63.8 ± 1.8 (60.1-67.6)	63.7 ± 2.16 (60.82-68.54)	64.0 (61.0-67.0)	60.0-65.0	62.0 ± 2.85 (58.0-68.0)	63.0 ± 2.22 (59.0-65.0)
DGO	10.36 ± 2.6 (5.0-8.0)	12 ± 1.97 (8.0-15.0)	–	–	9.6 ± 2.07 (75.0-13.0)	9.1 ± 0.94 (7.0-10.0)
Stylet length	22.7 ± 1.0 (21.0-25.0)	22.8 ± 0.83 (21.0-24.0)	24.6 (21.7-26.8)	23.0-26.0	28.3 ± 1.23 (26.0-30.0)	27.9 ± 0.97 (25.5-29.0)
Number of head annuli	4.0 ± 0.0 (4.0-4.0)	4.0 ± 0.32 (3.0-5.0)	4.0	4.0-5.0	–	–
Tail length	20.53 ± 2.2 (17.0-25.0)	20.65 ± 2.39 (17.0-26.0)	17.9	–	20.6 ± 2.8 (16.0-24.0)	14.7 ± 2.71 (9.0-18.0)
Number of tail annuli	10.0 ± 2.8 (7.0-17)	11.0 ± 1.01 (10.0-13.0)	10.0-14.0	6.0-12.0	13.4 ± 0.97 (12.0-15.0)	10.2 ± 2.25 (7.0-15.0)
Position of phasmid	6.0 ± 0.9 (4.0-7.0)	3.0 ± 1.01 (1.0-5.0)	9	4/2	–	–

**Note:** Measurements in μm; mean ± s.d. (range), except for L in mm.

**Table 4.** Morphometric data of *H. californicus* and reference populations.

Species	*H. californicus*	*H. californicus*	*H. californicus*	*H. californicus*	*H. californicus*
Locality/publication	Plantain, Delfina–Buenaventura–HC1 *n* = 12	Banana, Brazil HC2 *n* = 19	Van Den Berg and Heyns (1975) *n* = 25	Uzma et al. (2015) *n* = not defined	Sher (1966) *n* = 16
L	0.6 ± 0.05 (0.5-0.6)	0.6 ± 0.1 (0.5-0.7)	0.7 (0.6-0.8)	0.58-0.78	0.6 (0.5-0.7)
a	23.5 ± 1.0 (22.0-25.2)	24.6 ± 1.4 (21.5-26.6)	28.8 (23.4-35.0)	27-32	24.1 (21.1-26.2)
c	35.5 ± 4.7 (27.2-45.8)	34.9 ± 3.0 (30.3-40.2)	39.0 (30.8-53.0)	32.0-50.0	38.7 (31.2-48.1)
c′	1.0 ± 0.1 (0.8-1.1)	1.1 ± 0.1 (1.0-1.3)	1.1 (0.7-1.5)	0.8-1.3	1.2 (0.9-2.1)
V%	62.1 ± 1.7 (59.3-64.8)	65.6 ± 3.0 (61.3-75.5)	63.0 (60.0-64)	59-64	64 (60-67)
DGO	11.4 ± 1.2 (10.0-14.0)	11.4 ± 2.4 (8.0-14.0)	–	–	10 (8-14)
Stylet length	22.4 ± 0.9 (20.0-23.0)	23.5 ± 1.0 (21.0-25.0)	24.1 (22.8-27.2)	24-27	27 (26-29)
Number of head annuli	4.7 ± 0.7 (4.0-6.0)	4.2 ± 0.5 (4.0-6.0)	3.0-5.0	4	5-7
Tail length	15.9 ± 1.0 (13.0-19.0)	16.5 ± 1.5 (14.0-20.0)	17.9 (15.1-25.0)	–	16 (13-21)
Number of tail annuli	7.8 ± 1.1 (6.0-9.0)	7.8 ± 0.9 (6.0-9.0)	10.0-17.0	8	9 (6-12)
Position of phasmid	6.8 ± 1.3 (5.0-8.0)	6.0 ± 2.7 (2.0-8.0)	3.0-14.0	2	1-11

**Note:** Measurements in μm; mean ± s.d. (range), except for L in mm.

The species *H. californicus* was identified in a plantain crop from Colombia (Delfina–Buenaventura, Colombia) and a banana crop from Brazil (Minas Gerais). Morphologically, these populations presented a habitus post-mortem in a spiral, a hemispherical cephalic region with the shape of stylet knobs flattened anteriorly and rounded posteriorly, an irregular tail with a short sharply pointed or blunt ventral projection and males present (Fig. 2; Table 4).

**Figure 3:**
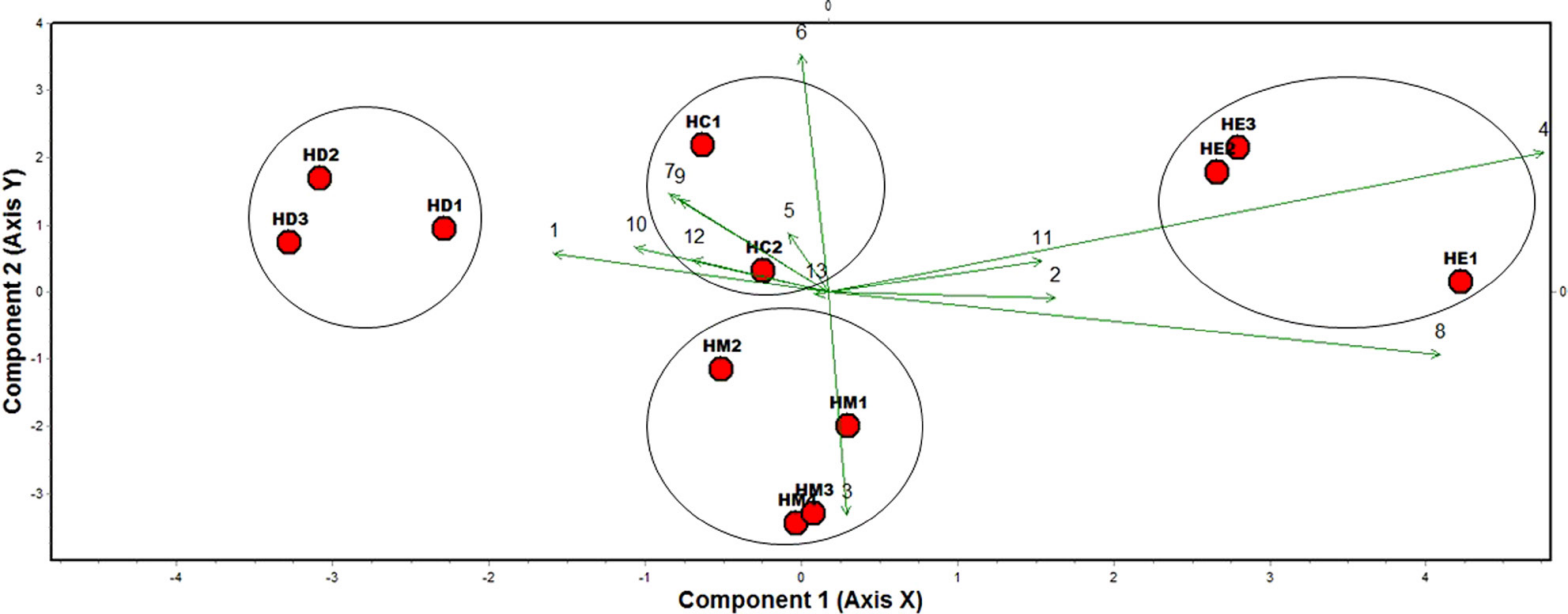
Populations of *H. multicinctus* (HM)*, H. dihystera* (HD)*, H. erythrinae* (HE), and *H. californicus* (HC) from Colombia and Brazil can be assigned to its corresponding species based on morphometric data. The two first axes of a principal components analysis (PCA) are shown. HM1 to HM2 from Colombia and HM3 to HM4 from Brazil; HD1 to HD3 from Colombia; HE1 to HE3 from Colombia; and HC1 from Colombia and HC2 from Brazil.

**Figure 4:**
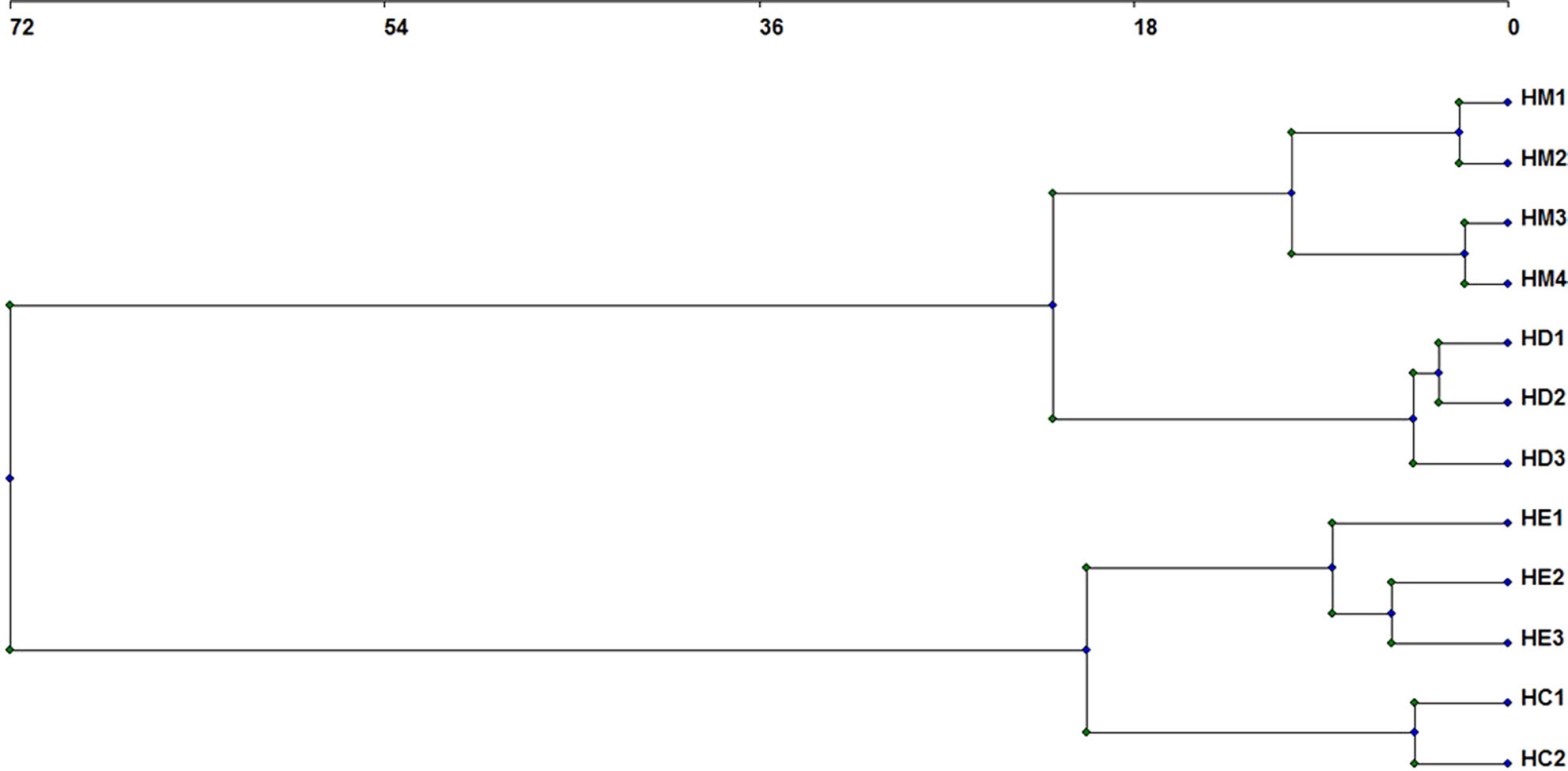
Dendrogram obtained by a conglomerates analysis to classify the Colombian and Brazilian populations of *H. multicinctus* (HM)*, H. dihystera* (HD)*, H. erythrinae* (HE), and *H. californicus* (HC). HM1 to HM2 from Colombia and HM3 to HM4 from Brazil; HD1 to HD3 from Colombia; HE1 to HE3 from Colombia; and HC1 from Colombia and HC2 from Brazil.

PCA and conglomerate analysis separated the species into four groups (*H. multicinctus, H. dihystera, H. erythrinae*, and *H. californicus*) (Figs. 3, 4). The principal components 1 to 4 have eigenvalues greater than or equal to 1 and explain 91.39% of variance (Table 5). However, PC1 and PC2 axes better separated the species. In PC1, the most discriminating variables were maximum body diameter, the number of tail annuli, and ratio c′, while in PC2 they were vulva position, anal body diameter, and tail length (Table 6).

**Table 5.** Eigenvalues and percent total variance accounted for each principal component.

Principal component	Eigenvalues	Cumulative total	% of Total variance	Cum. % of total variance
1	4.92	4.92	37.88	37.88
2	3.75	8.68	28.88	66.76
3	2.16	10.84	16.63	83.39
4	1.04	11.88	7.99	91.39
5	0.35	12.23	2.70	94.09
6	0.29	12.52	2.22	96.31
7	0.22	12.74	1.68	97.99
8	0.16	12.89	1.23	99.22
9	0.06	12.95	0.43	99.65
10	0.04	12.99	0.29	99.94
11	0.01	13	0.06	100
12	1.5128E–7	13	1.16369E–6	100
13	–4.65844E–10	13	–3.58342E–9	100

**Table 6.** Correlations between the first four component principals and the morphometric parameters of females in *Helicotylenchus* spp.

Vector	Diagnostic character	1	2	3	4
1	L	–0.25	0.21	0.02	0.66
2	a	0.09	–0.01	–0.57	0.35
3	c	–0.34	–0.33	–0.01	0.02
4	c′	*0.36*	0.28	–0.12	–0.11
5	V %	–0.06	*–0.48*	–0.13	0.01
6	DGO	–0.22	0.35	–0.25	–0.27
7	Stylet length	–0.31	–0.05	–0.39	–0.03
8	Number of head annuli	0.01	0.05	0.61	0.07
9	Number of tail annuli	*0.37*	0.07	–0.09	0.33
10	Position of phasmid	–0.29	0.31	–0.09	–0.40
11	Maximum body diameter	*–0.39*	0.22	0.02	0.15
12	Tail length	0.32	*0.35*	–0.10	–0.05
13	Anal body diameter	–0.24	*0.37*	0.17	0.22

**Note:** Key diagnostics for discriminating *Helicotylenchus* species are denoted in italic.

### Molecular identification of nematodes

In relation to the D2 to D3 expansion segment of ribosomal DNA, four consensus sequences were obtained for *H. multicinctus* with similarity of 99% with different sequences of this species deposited in the NCBI (KF443214, DQ328745, DQ328746, HM014290, HM014291, and HM014292). On the other hand, two consensus sequences were obtained for *H. dihystera*, which presented similarity of 99% with other sequences of this species already deposited in NCBI (HM014251, KF486503, HM014250, HM014245, HM014246, HM014247, HM014248, HM014249, KM506834, KM506835, and KM506836). Molecular identity of the sequence obtained from the individual morphologically identified as *H. erythrinae* was not confirmed, because there are no reference sequences of *H. erythrinae* in NCBI or any other molecular database. When BLAST was performed, no sequence deposited in NCBI presented similarity greater than or equal to 99% with the sequence obtained in this research for *H. erythrinae* and closer sequences were of *H. labiodiscinus* with a similarity of 90.85 to 91.03% (HM014293; HM014294; HM014295). For individuals identified morphologically as *H. californicus* were obtained four sequences that showed similarity of 99% with an isolate of *Helicotylenchus* sp. labeled as CD761, with accession numbers KM506844 and KM506845. The sequences obtained in this study for individuals identified morphologically as *H. erythrinae* and *H. californicus* are proposed as standard and reference populations until topotype specimens become available and were molecularly characterized. All sequences obtained in the present study were deposited in NCBI with accession numbers MT321729-MT321739 (Table 7).

**Table 7.** Information of sequences D2 to D3 of ribosomal DNA downloaded from GenBank and obtained in the present study for *Helicotylenchus*.

Species name	Location	Plant-host	GenBank accession number	Reference or source
*H. dihystera*	*Colombia, Córdoba, Quindío*	*Musa AAA (Banana)*	*MT321729; MT321730*	*Present study*
*H. dihystera*	USA, Florida, Ft Lauderdale	*Schefflera arboricola*	HM014245	Subbotin et al. (2011)
*H. dihystera*	USA, Florida, Goulds	Bromeliads	HM014246	Subbotin et al. (2011)
*H. dihystera*	USA, Florida, Ft Pierce	*Ficus*	HM014247	Subbotin et al. (2011)
*H. dihystera*	USA, Hawaii, Kawai	Grasses	HM014248	Subbotin et al. (2011)
*H. dihystera*	USA, Georgia	–	HM014249	Subbotin et al. (2011)
*H. dihystera*	USA, Hawaii, Kawai	Grasses	HM014250	Subbotin et al. (2011)
*H. dihystera*	USA, Georgia	–	HM014251	Subbotin et al. (2011)
*H. dihystera*	USA, California, Kern county	*Vitis vinifera*	KM506834	Subbotin et al. (2015)
*H. dihystera*	USA, Texas (plant shipment)	–	KM506835	Subbotin et al. (2015)
*H. dihystera*	USA, California, Merced	Grasses	KM506836	Subbotin et al. (2015)
*H. dihystera*	China, Fujian Province	*Musa* sp.	KF486503	Xiao et al. (2014)
*H. leiocephalus*	USA, Nebraska, Lincoln, Nine mile Prairie	*Amorpha canescens*	HM014268; HM014269	Subbotin et al. (2011)
*H. pseudorobustus*	USA, Kansas, Konza Prairie, Manhattan	*Koeleria pyramidata*	HM014266; HM014267	Subbotin et al. (2011)
*H. pseudorobustus*	New Zealand, MAF farm, Kaitoke	*Lolium perenne*, *Trifolium repens*	HM014278	Subbotin et al. (2011)
*H. pseudorobustus*	New Zealand, Rotorua	*Lolium perenne*	HM014279; HM014280	Subbotin et al. (2011)
*H. pseudorobustus*	China, Beijing	–	DQ328747	Subbotin et al. (2011)
*H. pseudorobustus*	USA, California, Fresno	–	DQ328748	Subbotin et al. (2011)
*H. pseudorobustus*	Italy, Ancona	–	DQ328750	Subbotin et al. (2011)
*H. pseudorobustus*	Germany, Münster, BBA glasshouse	–	DQ328751	Subbotin et al. (2011)
*Helicotylenchus* spIV-2	USA, Florida, Gainesville, Winter garden	*Calathea* sp.	KM506844	Subbotin et al. (2015)
*H. multicinctus*	*Colombia, Calarcá, Quindío*	*Musa AAA (Banana)*	*MT321731*	*Present study*
*H. multicinctus*	*Colombia, Rozo, Palmira, Valle del Cauca*	*Musa AAB (Plantain)*	*MT321732; MT321733; MT321734*	*Present study*
*H. multicinctus*	South Africa, Lambani	*Musa* sp.	HM014290; HM014291	Subbotin et al. (2011)
*H. multicinctus*	USA, Florida, Ft. Pierce	*Ficus benjamina*	HM014292	Subbotin et al. (2011)
*H. multicinctus*	Sudan	*Musa* sp.	DQ328745; DQ328746	Subbotin et al. (2011)
*H. multicinctus*	China, Fujian Province	*Musa* sp.	KF443214	Xiao et al. (2014)
*H. californicus*	*Colombia, La Delfina, Buenaventura, Valle del Cauca*	*Musa AAB (Plantain)*	*MT321735; MT321736; MT321737; MT321738*	*Present study*
*H. erythrinae*	*Colombia, La Celia, Risaralda*	*Musa AAB (Plantain)*	*MT321739*	*Present study*
*H. labiodiscinus*	USA, Kansas, Manhattan	*Poa pratensis*	HM014293	Subbotin et al. (2011)
*H. labiodiscinus*	USA, Kansas, Manhattan	*Andropogon gerardii*	HM014294	Subbotin et al. (2011)
*H. labiodiscinus*	USA, Kansas, Manhattan	*Schizachyrium scoparium*	HM014295	Subbotin et al. (2011)
*H. martini*	South Africa, Lambani	Grasses	HM014304; HM014305	Subbotin et al. (2011)
*H. brevis*	South Africa, North West Province	*Solanum mauritianum*	HM014299; HM014300	Subbotin et al. (2011)
*H. vulgaris*	Italy, Ancona	–	DQ328759; DQ328760; DQ328761	Subbotin et al. (2011)
*H. vulgaris*	USA, Kansas, University of Arkansas	–	FJ485650	Subbotin et al. (2011)

**Note:** Information of new sequences obtained in the present study are marked in italic.

### Phylogenetic relationships of nematodes

**Figure 5:**
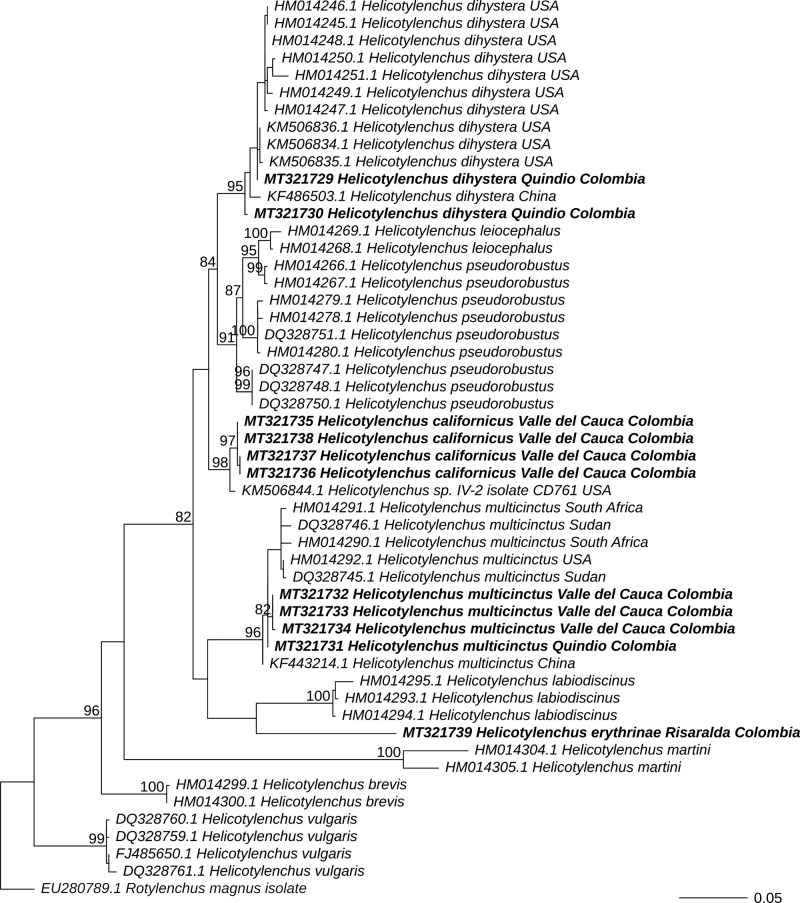
Maximum likelihood phylogeny of *Helicotylenchus*. The tree was estimated using the D2 to D3 expansion segment of 28S RNAr and 250 bootstraps under GTR + Γ model. The outgroup (*Rotylenchus magnus*) is shown in gray font; the sequences that were obtained in this study appear in bold typeface. Values at the nodes represent the bootstrap support. Were provided bootstrap support for nodes with values > 80. The scale represents the number of substitutions per site.

**Figure 6:**
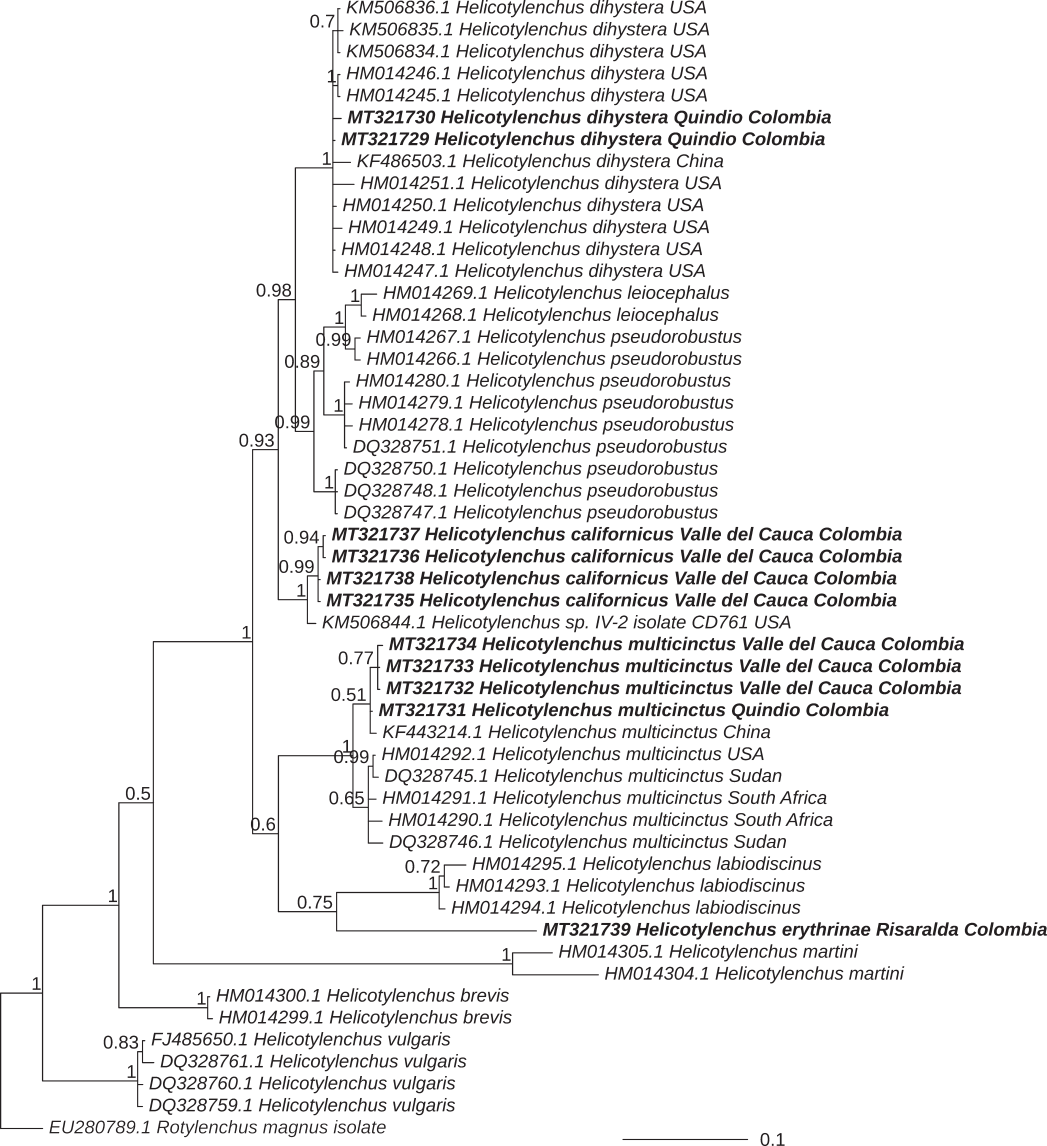
Bayesian phylogeny of *Helicotylenchus* based on D2 to D3 expansion segment of 28S RNAr. The phylogeny is a consensus tree from a posterior distribution of 1,600 trees that were inferred under GTR + Γ model in MrBayes. The outgroup (*Rotylenchus magnus*) is shown in gray font; the sequences that were obtained in this study appear in bold typeface. Values at the nodes represent the posterior probability. Were provided posterior probabilities for nodes with values > 0.5. The scale represents the number of substitutions per site.

The evolutionary relationships of 51 sequences of the D2 to D3 expansion segment of ribosomal DNA for *Helicotylenchus*, including those obtained in the present study (Table 7), are depicted in Figures 5 and 6. The sequences of *H. multicinctus* obtained in this research clustered in the same clade with other sequences of this species, including some populations from *Musa* sp. plantations, such as DQ328745 and DQ328746 from Sudan, HM014290 and HM014291 from Lambani, South Africa, and KF443214 from Fujian, China. Monophyly of this species was recovered both with maximum likelihood (bootstrap support or BS = 96%) and Bayesian inference (posterior probability of PP = 1). Likewise, the sequences of *H. dihystera* from Colombia obtained in this research, grouped in the same clade with other sequences of that species, including HM014248 and HM014250 from grasses in Hawaii and KF486503 from *Musa* sp. in Fujian, China (BS = 95%, PP = 1). The sequence of *H. erythrinae* grouped with the clade of *H. labiodiscinus*, although the support for this group was weak (BS = 63%, PP = 0.75). Finally, the sequences of *H. californicus* formed a clade with a sequence of *Helicotylenchus* sp. isolate CD761 from *Calathea* in USA (KM506844) (BS = 98%, PP = 1). These results support the presence of four species of *Helicotylenchus* in the *Musa* crops that were sampled.

## Discussion

A total of 10 *Helicotylenchus* populations associated with *Musa* spp. crops were identified to the species level in the present study: four *H. multicinctus*, two *H. dihystera*, two *H. californicus*, and two *H. erythrinae*. All species identified in this study occur in Colombia, while only two species were registered in Brazil (*H. multicinctus* and *H. californicus*). Morphometric measurements recorded for these species closely resemble the type and reference populations (Golden, 1956; Sher, 1961; Van Den Berg and Heyns, 1975; Krall, 1990; Mizukubo et al., 1992; Wount and Yeates, 1994; Uzma et al., 2015).

Although it is reported that morphological and morphometric identification of species in *Helicotylenchus* is a difficult task because many species share very similar diagnostic characters and overlapping morphometrics, the populations of *Helicotylenchus* studied associated with *Musa* spp. were satisfactorily identified to the species level through morphological and morphometric evidence. PCA and conglomerate analysis clearly separated the four species identified, confirming the utility of morphological and morphometric data but also of multivariate statistical analysis to discriminate among species of the genus (Fortuner and Maggenti, 1991; Subbotin et al., 2015; Uzma et al., 2015). In accordance with PCA, the variables associated with the tail are diagnostic characters powerful enough to discriminate between or to separate among *H. multicinctus*, *H. dihystera*, *H. californicus*, and *H. erythrinae*, which could be useful for preparing a dichotomous key for identification of *Helicotylenchus* species in Colombia (Mizukubo et al., 1992; Uzma et al., 2015).

The diagnostic characters: number of tail annuli, ratio c′, maximum body diameter, tail length, anal body diameter, and vulva position separated the species identified in this study in accordance with PCA and conglomerate analysis. In various publications, tail length and DGO have been suggested by their discriminate values among *Helicotylenchus* species (Perry et al., 1959; Fortuner et al., 1984). Additionally, intraspecific variability at the morphometric level was observed in the four species identified in this study, due to the various morphological and morphometric unconstant characters with a coefficient of high variability within of *Helicotylenchus* species (Fortuner et al., 1981; Fortuner, 1984).

Different morphological and morphometric characters were constants within the species identified, as habitus, ratio a and V as reported for various species of *Helicotylenchus* (Fortuner, 1984). Variation in the shape of the tail was registered in females of the Colombian population of *H. californicus*. This species is characterized by showing individuals with short ventral projections and sharply pointed or blunt tails (Van Den Berg and Heyns, 1975; Krall, 1990). Variability in the shape of the tail to the intraspecific level has been recorded for various species of *Helicotylenchus* (Fortuner, 1979).

Molecular analysis supported the presence of *H. multicinctus, H. dihystera, H. californicus*, and *H. erythrinae* in *Musa* spp. crops from Colombia. Interestingly, in the phylogenetic tree, Colombian sequences of *H. multicinctus* and *H. dihystera* clustered in the same clade with sequences of nematodes isolated from banana crops in Africa and China, respectively (Subbotin et al., 2011; Xiao et al., 2014). The sequences obtained for *H. californicus* grouped with an isolate of *Helicotylenchus* from *Calathea* not identified to the species level and labeled as CD761 in Subbotin et al. (2015) (Table 7). Recently, Fortuner et al. (2018) identified isolate CD761 as *H. pseudorobustus* using morphological and morphometric analysis. However, our populations are similar to preliminary reports of *H. californicus* (Van Den Berg and Heyns, 1975; Krall, 1990). Unfortunately, there are no sequences of reference for *H. erythrinae* deposited in any of the databases of genes in the world for comparison. Therefore, the present study is reporting the first sequences of *H. californicus* and *H. erythrinae* associated to *Musa* spp. in a public database.

The four species identified in this study, *H. multicinctus, H. dihystera, H. californicus*, and *H. erythrinae*, have been reported in *Musa* spp. crops around the world (Campos et al., 1987; Araya and De Waele, 2004; Karakas, 2007; Dias-Arieira, 2010; Ravichandra, 2014; Xiao et al., 2014; Lara et al., 2016; Tzortzakakis et al., 2017). However, this is the first report of these species for Musaceae crops in Colombia through integrative taxonomy. Additionally, this research confirmed that *Helicotylenchus* species occur individually or in a mixture in Musaceae crops in the studied zones, which has been documented in the past in other production zones of the world (Araya and De Waele, 2004; Dias-Arieira, 2010; Roy et al., 2014; Khan and Hasan, 2010; Ravichandra, 2014; Daneel et al., 2015).

In relation to *H. multicinctus*, it is considered the most limiting species of *Helicotylenchus* in plantain and banana. It was present in the *Musa* crop production of both Colombia and Brazil, confirming the wide distribution of this plant-parasitic nematode, which also has been previously registered in Puerto Rico, Vietnam, India, Mexico, and Greece (Ngoc Chau et al., 1997; Ravichandra, 2014; Lara et al., 2016; Tzortzakakis et al., 2017). The presence of *H. multicinctus* in the different production zones, Quindío and Valle del Cauca, suggests a wide spread of this nematode in Colombia, directly related with the dispersion of contaminated seedlings between production zones, but also to environmental conditions that have favored the establishment of this species (altitude, soil type, and nutrient availability for the plant) (Nath et al., 1998; Speijer and Ssango, 1999; Araya et al., 2011; Godefroid et al., 2017).

## References

[ref001] Araya M. and De Waele D. 2004 Spatial distribution of nematodes in three banana (*Musa* AAA) root part considering two root thickness in three farm management systems. Acta Oecologica 26:137–148.

[ref002] Araya M., Serrano E. and Vargas A. 2011 Relación entre el contenido de nutrientes en suelo y raíces de banano (*Musa* AAA) con el peso de raíces y número de nematodos. Fitosanidad 15:163–177.

[ref003] Barekye A., Kashaija I. N., Tushemereirwe W. K. and Adipala E. 2000 Comparison of damage levels caused by *Radopholus similis* and *Helicotylenchus multicinctus* on bananas in Uganda. Annals of Applied Biology 137:273–278.

[ref004] Boag B. and Shamim Jairajpuri M. 1985 *Helicotylenchus scoticus* n. sp. and a conspectus of the genus *Helicotylenchus* Steiner, 1945 (Tylenchida: Nematoda). Systematic Parasitology 7:47–58.

[ref005] Campos V., D´arc R. and Ferreira V. 1987 Nematóides parasitos de grandes culturas identificados em localidades de Minas Gerais e S*ã*o Paulo. Nematologia Brasileira 11:226–232.

[ref006] Castillo P. and Gómez–Barcina A. 1993 Plant–parasitic nematodes associated with tropical and subtropical crops in southern Spain. Nematology Mediterranean 21:45–47.

[ref007] Crozzoli R. 2009 “Nematodes of tropical fruit crops in Venezuela”, In Ciancio A. and Mukerji K. G. (Eds), Integrated management of fruit crops and forest nematodes Springer, India, 63–83.

[ref008] Daneel M., De Jager K., Van Den Bergh I., De Smet M. and De Waele D. 2015 Occurrence and pathogenicity of plant–parasitic nematodes on commonly grown banana cultivars in South Africa. Nematropica 45:118–127.

[ref009] De Ley P., Felix M. A., Frisse L. M., Nadler S. A., Sternberg P. W. and Thomas W. K. 1999 Molecular and morphological characterisation of two reproductively isolated species with mirror–image anatomy (Nematoda: Cephalobidae). Nematology 1:591–612.

[ref010] Dias-Arieira C. 2010 Fitonematoides associados a frutíferas na região Noroeste do Paraná, Brasil. Revista Brasileira de Fruticultura 32:1064–1071.

[ref011] Fortuner R. 1979 Morphometrical variability in *Helicotylenchus* Steiner, 1945. I. The progeny of a single female. Revue de Nématologie 2:197–202.

[ref012] Fortuner R. 1984 Morphometrical variability in *Helicotylenchus* Steiner, 1945. 6: value of the characters used for specific identification. Revue de Nématologie 7:245–264.

[ref013] Fortuner R. and Maggenti A. 1991 A statistical approach to the objective differenciation of *Hirschmanniella oryzae* from H. belli (Nemata: Pratylenchidae). Revue de Nématologie 114:165–180.

[ref014] Fortuner R., Merny G. and Roux C. 1981 Morphometrical variability in *Helicotylenchus* Steiner, 1945. 3. Observations on African populations of *Helicotylenchus dihystera* and considerations on related species. Revue de Nématologie 4:235–260.

[ref015] Fortuner R., Maggenti A. and Whittaker L. 1984 Morphometrical variability in *Helicotylenchus* Steiner, 1945. 4: Study of field populations of *H. pseudorobustus* and related species. Revue de Nématologie 7:121–135.

[ref016] Fortuner R., Louis P. and Geniet D. 2018 On the morphometric identity of populations of *Helicotylenchus pseudorobustus* (Steiner, 1914) Golden, 1956 (Tylenchida: Hoplolaimidae). Nematology 20:423–439.

[ref017] Godefroid M., Tixier P., Chabrier C., Djigal D. and Quénéhervé P. 2017 Associations of soil type and previous crop with plant–feeding nematode communities in plantain agrosystems. Applied Soil Ecology 113:63–70.

[ref018] Golden A. (1956), “Taxonomy of the spiral nematode (*Rotylenchus* and *Helycotylenchus*), and the developmental stages and host–parasite relationship of *R. buxophilus* n. sp., attacking boxwood, Bulletin A–85”, Maryland Agricultural Experiment Station, Baltimore.

[ref019] Goodey T. 1940 On *Anguillulina multicincta* (Cobb) and other species of *Anguillulina* associated with roots of plants. Journal of Helminthology 18:21–38.

[ref020] Guzmán-Piedrahita Ó. 2011 Importancia de los nematodos espiral, *Helicotylenchus multicinctus* (COBB) Golden y *H. dihystera* (COBB) Sher, en banano y plátano. Agronomía 19:19–32.

[ref021] Guzmán-Piedrahita Ó. and Cataño–Zapata J. 2004 Reconocimiento de nematodos fitopatógenos en plátanos dominico hartón (*Musa* AAB Simmonds), África, Fhia–20 y Fhia–21 en la granja Montelindo, municipio de Palestina (Caldas), Colombia. Revista de la Academia Colombiana de Ciencias Exactas, Físicas y Naturales 28:295–301.

[ref023] Kamira M., Hauser S., Van Asten P., Coyne D. and Talwana H. L. 2013 Plant parasitic nematodes associated with banana and plantain in eastern and western Democratic Republic of Congo. Nematropica 43:216–225.

[ref024] Karakas M. 2007 Life cycle and mating behavior of *Helicotylenchus multicinctus* (Nematoda: Hoplolaimidae) on excised *Musa cavendishii* roots. Biologia Bratislava 62:320–322.

[ref025] Katoh K., Misawa K., Kuma K. and Miyata T. 2002 MAFFT: a novel method for rapid multiple sequence alignment based on fast Fourier transform. Nucleic Acids Research 30:3059–3066. 12136088 10.1093/nar/gkf436 PMC135756

[ref026] Kearse M., Moir R., Wilson A., Stones S., Cheung M., Sturrock S., Buxton S., Cooper A., Markowitz S., Duran C., Thierer T., Ashton B., Meintjes P. and Drummond A. 2012 Geneious basic: an integrated and extendable desktop software platform for the organization and analysis of sequence data. Bioinformatics 28:1647–1649. 22543367 10.1093/bioinformatics/bts199 PMC3371832

[ref027] Khan M. and Hasan M. 2010 Nematode diversity in banana rhizosphere from west Bengal, India. Journal of Plant Protection Research 50:263–267.

[ref028] Krall E. 1990 Root parasitic nematodes family Hoplolaimidae Paul Press, New Delhi.

[ref029] Lara S., Núñez A., López–Lima D. and Carrión G. 2016 Nemátodos fitoparásitos asociados a raíces de plátano (*Musa acuminata* AA) en el centro de Veracruz, México. Revista Mexicana de Fitopatología 34:116–130.

[ref030] McSorley R. and Parrado J. L. 1986 *Helicotylenchus multicinctus* on bananas: an international problem. Nematropica 16:73–91.

[ref031] Miller M. A., Pfeiffer W. and Schwartz T. 2010 Creating the CIPRES Science Gateway for inference of large phylogenetic trees. Gateway Computing Environments Workshop, pp. 1–8.

[ref032] Mizukubo T., Toida Y., and Keereewan 1992 A survey of the nematodes attacking crops in Thailand I genus *Helicotylenchus* Steiner, 1945. Japanese Journal of Nematology 22:26–36.

[ref033] Múnera G. E., Bert W. and Decraemer W. 2009 Morphological and molecular characterization of *Pratylenchus araucensis* n. sp. (Pratylenchidae), a root–lesion nematode associated with *Musa* plants in Colombia. Nematologica 11:799–813.

[ref034] Nath R., Mukherjee B. and Dasgupta M. 1998 Population behaviour of *Helicotylenchus multicinctus* in soil and roots of banana in Tripura, India. Fundamental Applied. Nematology 21:353–358.

[ref035] Ngoc Chau N., Vu Thanh N., De Waele D. and Geraert E. 1997 Plant–parasitic nematodes associated with banana in Vietnam. International Journal of Nematology 7:122–126.

[ref036] Orbin D. 1973 Histopathology of soybean roots infected with *Helicotylenchus dihystera* . Journal of Nematology 5:37–40. 19319293 PMC2619959

[ref037] Perry V., Darling H. and Thorne G. 1959 Anatomy, taxonomy and control of certain spiral nematodes attacking blue grass in Wisconsin. Research bulletin/College of Agricultural and Life Sciences, Research Division, 207, University of Wisconsin, Wisconsin.

[ref038] Posada D. 2008 jModelTest: phylogenetic model averaging. Molecular Biology and Evolution 25:1253–1256. 18397919 10.1093/molbev/msn083

[ref039] Ravichandra N. G. 2014 “Nematode diseases of horticultural crops”, In Ravichandra N. G. (Ed.), Horticultural nematology Springer, India, 127–205.

[ref040] Ronquist F., Teslenko M., van der Mark P., Ayres D., Darling A., Höhna S., Larget B., Liu L., Suchard M. and Huelsenbeck J. P. 2012 MrBayes 3.2: efficient Bayesian phylogenetic inference and model choice across a large model space. Systematic Biology 61:539–542. 22357727 10.1093/sysbio/sys029 PMC3329765

[ref041] Roy K., Roy S., Sarkar S., Rathod A. and Pramanik A. 2014 Diversity of migratory nematode endoparasites of banana. Journal of Crop and Weed 10:375–391.

[ref042] Selvaraj S., Ganeshamoorthi P., Anand T., Raguchander T., Seenivasan N. and Samiyappan R. 2014 Evaluation of a liquid formulation of *Pseudomonas fluorescens* against *Fusarium oxysporum* f. sp. *cubense* and *Helicotylenchus multicinctus* in banana plantation. Biocontrol 59:345–355.

[ref043] Sher S. 1961 Revisión of the Hoplolaiminae (Nematoda). I. Classification of nominal genera and nominal species. Nematologica 6:155–169.

[ref044] Sher S. 1966 Revisión of the Hoplolaiminae (Nematoda) VI. *Helicotylenchus* Steiner, 1945. Nematologica 12:1–56.

[ref045] Siddiqi M. 1972 *Helicotylenchus dihystera*. C.I.H. Description of plant–parasitic nematodes. Set 1, No. 9. Agricultural Bureaux, Farnham, Royal, UK.

[ref047] Singh S. K., Hodda M. and Ash J. 2013 Plant–parasitic nematodes of potential phytosanitary importance, their main hosts and reported yield losses. Bulletin OEPP/EPPO 43:334–374.

[ref048] Speijer P. R. and Ssango F. 1999 Evaluation of *Musa* host plant response using nematode densities and damage indices. Nematropica 29:185–192.

[ref049] Stamatakis A. 2014 RAxML version 8: a tool for phylogenetic analysis and post–analysis of large phylogenies. Bioinformatics 30:1312–1313. 24451623 10.1093/bioinformatics/btu033 PMC3998144

[ref050] Subbotin S., Inserra R., Marais M., Mullin P., Powers T., Roberts P., Van Den Berg E., Yeates G. and Baldwin J. 2011 Diversity and phylogenetic relationships within the spiral nematodes of *Helicotylenchus* Steiner, 1945 (Tylenchida: Hoplolaimidae) as inferred from analysis of the D2–D3 expansion segments of 28S rRNA gene sequences. Nematology 13:333–345.

[ref051] Subbotin S., Vovlas N., Yeates G., Hallmann J., Kiewnick S., Chizhov V., Manzanilla–López R., Inserra R. and Castillo P. 2015 Morphological and molecular characterisation of *Helicotylenchus pseudorobustus* (Steiner, 1914) Golden, 1956 and related species (Tylenchida: Hoplolaimidae) with a phylogeny of the genus. Nematology 17:27–52.

[ref052] Torrado-Jaime M. and Castaño-Zapata J. 2009 Incidencia de nematodos en plátano en distintos estados fenológicos. Agronomía Colombiana 27:237–244.

[ref053] Tzortzakakis E., Cantalapiedra–Navarrete C., Castillo P., Palomares-Rius J. and Archidona-Yuste A. 2017 Morphological and molecular identification of *Longidorus euonymus* and *Helicotylenchus multicinctus* from the rhizosphere of Grapevine and banana in Greece. Journal of Nematology 49:233–235. 29062145 PMC5644915

[ref054] Uzma I., Nasira K., Firoza K. and Shahina F. 2015 Review of genus Helicotylenchus Steiner, 1945 (Nematoda: Hoplolaimidae) with update diagnostic compendium. Pakistan Journal of Nematology 33:115–160.

[ref055] Van Den Berg E. and Heyns J. 1975 South African Hoplolaiminae. 4. The genus *Helicotylenchus* Steiner, 1945. Phytophylactica 7:35–52.

[ref056] Van Den Berg E., Marais M., Gaidashova S. and Tiedt L. 2003 Hoplolaimidae Filip’ev, 1934 (Nemata) from Rwandan banana fields. African Plant Protection 9:31–42.

[ref057] Villegas C. 1989 “Reconocimiento de nematodos en plátano Dominico–Hartón enano Musa AAB”, In Cayón D. and Salazar F. (Eds), Resumenes análiticos de la investigación sobre el plátano en Colombia Corpoica–Inibap–Asiplat, Colombia, 275.

[ref058] Wount W. and Yeates G. 1994 *Helicotylenchus* species (Nematoda: Tylenchida) from native vegetation and undisturbed soils in New Zealand. New Zealand Journal of Zoology 21:213–224.

[ref059] Xiao Y., Zhou X. and Zhang S. 2014 Identification of Helicotylenchus and Hoplolaimus species parasitized banana in Fujian, China. Journal of Fujian Agriculture and Forestry University 43:573–578.

[ref060] Zuñiga G., Ortiz R. and Varón de Agudelo F 1979 Nematodos asociados con el cultivo del plátano (*Musa* AAB ó ABB) en el Valle del Cauca. Fitopatología colombiana 8:40–52.

